# The Liver Cancer Immune Microenvironment: Emerging Concepts for Myeloid Cell Profiling with Diagnostic and Therapeutic Implications

**DOI:** 10.3390/cancers15051522

**Published:** 2023-02-28

**Authors:** Konstantinos Arvanitakis, Ioannis Mitroulis, Antonios Chatzigeorgiou, Ioannis Elefsiniotis, Georgios Germanidis

**Affiliations:** 1 First Department of Internal Medicine, AHEPA University Hospital, Aristotle University of Thessaloniki, 54636 Thessaloniki, Greece; 2 Basic and Translational Research Unit (BTRU) of Special Unit for Biomedical Research and Education (BRESU), Faculty of Health Sciences, School of Medicine, Aristotle University of Thessaloniki, 54636 Thessaloniki, Greece; 3 First Department of Internal Medicine, University Hospital of Alexandroupolis, Democritus University of Thrace, 68100 Alexandroupolis, Greece; 4 Department of Physiology, Medical School, National and Kapodistrian University of Athens, 11527 Athens, Greece; 5 University Department of Internal Medicine, General and Oncology Hospital of Kifisia Agioi Anargyroi, 14564 Athens, Greece

## 1. Introduction

Hepatocellular carcinoma (HCC) is one of the leading causes of cancer-related deaths worldwide. To date, systemic treatment for patients with unresectable or advanced disease was composed of sorafenib and other multikinase inhibitors, with limited efficacy and high toxicity. Nevertheless, immune checkpoint inhibitors (ICIs) have greatly broadened the treatment landscape of unresectable HCC [1]. Specifically, the IMbrave150 trial demonstrated that in patients with unresectable HCC, combined treatment with the programmed-death ligand-1 (PD-L1) inhibitor, atezolizumab, alongside the vascular endothelial growth factor (VEGF) inhibitor, bevacizumab, prolonged the median overall survival (OS) to 19.2 months as compared to sorafenib treatment alone [2]. However, around 20–25% of patients exhibit complete primary resistance to atezolizumab, plus bevacizumab, indicating that the identification of patients who might gain the most from this therapy is crucial.

To date, there are no definite biomarkers in HCC that can accurately predict response or resistance to ICIs, while as the HCC treatment regimens have shifted towards immunotherapy, the identification of potent predictive and prognostic biomarkers has attracted attention. Although HCC formation has been attributed to certain viral or non-viral causes, nonalcoholic steatohepatitis (NASH), is a major driver of HCC as well [3]. Recent data suggest that NASH-related HCC might have decreased sensitivity to immunotherapy, due to the presence of CD8+ T cells and, especially, of the hepatic steatosis-induced CXCR6+ subset that was correlated with hepatocyte injury and potentiated the pathogenesis of NASH-related HCC via the secretion of pro-inflammatory cytokines and direct hepatocyte killing, mediated by the tumor necrosis factor (TNF) [4]. Moreover, local and systemic inflammation are considered hallmarks of cancer, and they have a pivotal role in HCC pathogenesis and progression [5]. An increased peripheral blood absolute neutrophil count and an elevated neutrophil to lymphocyte ratio (NLR ≥ 5) are considered markers of advanced disease, poor prognosis, and poor response to treatment with hepatic resection, transplantation, locoregional therapy, and tyrosine kinase inhibitors in patients with HCC. Indeed, systemic inflammation measured by NLR is independently a negative prognostic factor for patients with HCC under ICI therapy [6]. The measurement of the NLR across various time points could provide insight into how different values of this inflammatory marker could accurately predict patient response to systemic therapy, patient outcomes, or the development of adverse events (AEs).

## 2. TANs and TAMs in the Immune Microenvironment of HCC

The tumor immune microenvironment (TIME), being heterogeneous and comprised of a multitude of immune and stromal cells, is an essential factor that leads to tumor metastasis and relapse, as well as resistance to therapy, whereas the way in which different TIME cell subtypes are connected with the clinical relevance in liver cancer remains unclear [7]. Indeed, cells of innate and adaptive immunity coexist and interact within the liver microenvironment during HCC, especially in the case of NASH, during which chronic hepatic inflammation preexists the emergence of HCC [8]. As far as the innate immune cells are concerned, in a mouse model of NASH-related HCC, neutrophils were shown to predominantly increase in the course of the disease, in comparison to other immune cells [9]. Indeed, in humans, high numbers of tumor-associated neutrophils (TANs) are a biomarker of poor prognosis in HCC and various other cancers [10]. Nevertheless, whether TANs, either within the HCC TIME or in a peritumoral hepatic location, account for this association remains unclear. However, these results should be carefully assessed, since they derive from HCC patients undergoing liver resection or liver transplantation, and it is typical for such patients to present with early, localized disease with preserved performance status and liver function. In countries with a low prevalence of viral hepatitis and a high prevalence of nonalcoholic fatty liver disease (NAFLD), approximately 15% of patients with HCC present with early disease and are considered candidates for curative resection. Consequently, the results of the aforementioned studies regarding TANs, might not be similar and comparable for individuals with more advanced HCC that are usually candidates for systemic treatment and account for the majority of patients.

Neutrophils have considerable phenotypic plasticity and can exist in both tumor-promoting (TAN2) and tumor-suppressing (TAN1) states. Neutrophils may also have the ability to influence ICIs therapy. Recent data report that CXCR2+ neutrophils were found in human NASH and within the tumor of both human and mouse models of NASH-related HCC. The resistance of NASH-related HCC to anti-PD1 therapy is being overcome by co-treatment with a CXCR2 small molecule inhibitor, with evidence of reduced tumor burden and extended survival [11]. Anti-PD1 and CXCR2 inhibitors combine to selectively reprogram TANs from a protumor to an antitumor phenotype, which unlocks their potential for cancer therapy. The ability of CXCR2 antagonism to combine with ICI therapy in order to lead to enhanced therapeutic benefit in NASH-related HCC (and potentially in HCC related to other aetiologies) warrants further clinical investigation. Along the same line, a recent animal study added that ferroptosis, caused by a tumor-suppressive immune response, is characterised by a CXCL10-dependent infiltration of cytotoxic CD8+ T cells, which at the same time was counterbalanced by a PD-L1 upregulation on tumor cells, as well as by a marked myeloid-derived suppressor cell (MDSC) infiltration. A triple combination of a ferroptosis-inducing agent, a CXCR2 inhibitor, and an anti-PD1, greatly improved the survival of wild-type mice with liver tumors [12].

**Figure 1:**
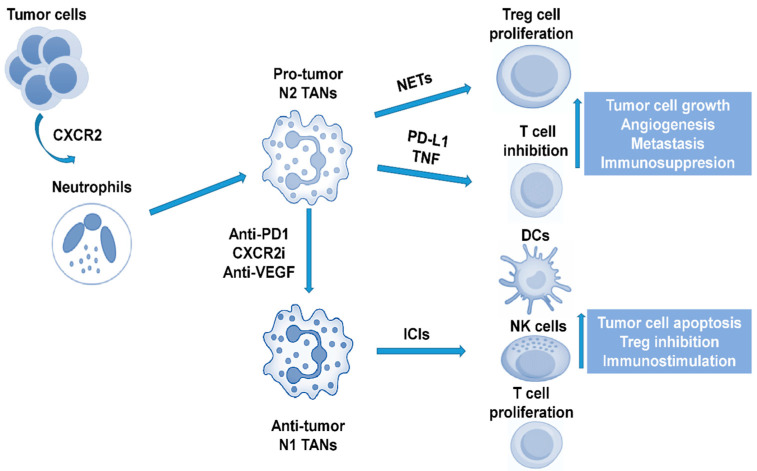
The role of neutrophils in the liver cancer immune microenvironment. CXCR2: C-X-C motif chemokine receptor 2; TAN: tumor-associated neutrophil; PD1: programmed cell death protein 1; CXCR2i: C-X-C motif chemokine receptor 2 inhibitor; NET: neutrophil extracellular trap; PD-L1: programmed death-ligand 1; ICI: immune checkpoint inhibitor; Treg: regulatory T; DC: dentritic cell; NK: natural killer.

Furthermore, another important study [13], by integrated analyses on molecular correlates of clinical response and resistance to atezolizumab in combination with bevacizumab in advanced patient samples of HCC collected within the phase Ib GO30140 and the phase III IMbrave150 trials, identified key molecular correlates of the combination therapy and highlighted that anti-VEGF might synergize with anti-PD-L1 by targeting angiogenesis, regulatory T-cells (Tregs) proliferation and myeloid cell inflammation. The presence of preexisting T-cell immunity is a key phenotypic characteristic that correlates with the response to atezolizumab plus bevacizumab. TIME potentiating the enrichment of the effector T-cell response over immunosuppressive Tregs identified patients that achieved significantly improved overall survival from the aforementioned combination. These findings were further validated by analyses of paired pre- and post-treatment biopsies, in situ analyses, and in vivo mouse models. Recent studies have also revealed the critical role of antigen non-specific auto-aggressive CD8+ T cells in instigating liver damage and promoting liver cancer in human NASH [14]. In addition, hepatic CD8+ PD1+ CXCR6+ T cells of humans with NASH, as well as neutrophil extracellular traps (NETs), contributed to the development of NASH-related HCC by promoting Treg differentiation, thus suppressing HCC immune surveillance [9,15] (Figure 1).

Through in vitro induction of TANs and ex vivo analyses of human TANs, a recent study also showed that CCL4+ TANs can recruit macrophages and that PD-L1 + TANs can suppress T cell cytotoxicity [16]. Monocytes are recruited into the tumor site by the release of tumoral and stromal chemokines, such as CCL2 and CCL15. Monocytes can be polarized into different subtypes such as CD14+, CCR1+, and CD14+ [7], while macrophages are in the epicentre of the molecular pathways regulating NASH-related HCC pathogenesis [17]. All of these subtypes promote a strong immunosuppressive environment with the expression of ICIs (PD-L1/2, B7-H3, and TIM3) and cytokines (IL-10, CXCL2, and CXCL8), inhibiting natural killer (NK) cytotoxicity, inducing Tregs. They also interact with neutrophils to promote tumor invasiveness through the oncostatin M pathway. A way to control tumorigenesis through monocytes would be through the prevention of their recruitment to the tumor site by inhibiting the CCL15 pathway, via blockade of their polarization by the inhibition of the p38 pathway, or via repressing the IL-6 pathway in order to prevent the formation of Tregs. The CD68 marker is commonly used for liver tumor-associated macrophage (TAM) localization and distribution, while the expression levels of CD86 (M1), CD163 (M2), and CD206 (M2) are used to distinguish between M1-like (inflammatory) and M2-like (anti-inflammatory) macrophages in vitro [18]. Collectively, these data show that non-viral HCC, and particularly NASH-related HCC, might be less responsive to immunotherapy, at least partially due to the presence of TANs and TAMs in the TIME of HCC.

## 3. Myeloid Cells and HCC Profiling

However, an important unmet clinical need is demonstrated by the lack of accurate biomarkers that can influence therapeutic choices. This unfulfilled need is being met by very few novel studies that incorporate multiregional single cell-dissection landscape of tumor and immune cells in HCC with the sole purpose of shedding light on the biological tumor characteristics and identifying potential tumor and blood biomarkers, in order to categorize specific groups of TIME and identify patients who might have a benefit from a specific treatment option. In brief, the combination of two single-cell RNA sequencing technologies [19], produced transcriptomes of CD45+ immune cells for HCC patients from five immune-relevant sites, and demonstrated an aggregate of LAMP3+ dendritic cells (DCs) that could modulate different subtypes of lymphocytes. Moreover, TAMs were correlated with poor prognosis, and the authors provided evidence of the inflammatory role of SLC40A1 and GPNMB in those cells.

Additionally, Sun et al. [20], by performing single-cell profiling in relapsed HCC, remarkably found that CD8+ T cells in recurrent tumors overexpressed KLRB1 (CD161) and displayed an innate-like low cytotoxic state, with low clonal expansion, unlike the classical exhausted state observed in primary HCC. The enrichment of these cells was associated with a worse prognosis. In addition, by performing multiregional single-cell RNA sequencing (scRNA-seq) analysis, Ma et al. [21], identified and further validated the cellular dynamics of malignant cells and their communication networks with tumor-associated immune cells in terms of ligand-receptor interaction pairs associated with unique transcriptome. These molecular networks of malignant ecosystems, may open a path for therapeutic exploration. Very recently, too, the first proteogenomic characterization of hepatitis B virus (HBV)-related HCC using paired tumor and adjacent liver tissues from 159 patients was performed by Gao Q et al. [22], and two prognostic biomarkers, PYCR2 and ADH1A, which were related to proteomic subgrouping and were involved in HCC metabolic reprogramming, were identified. CTNNB1 and TP53 mutation-associated signaling and metabolic profiles were revealed, among which, mutated CTNNB1-associated ALDOA phosphorylation was demonstrated to promote glycolysis and cell proliferation.

In a molecular study of HCC in patients with NASH, NASH-related HCCs were characterized by bile and fatty acid signaling, oxidative stress, and inflammation, and demonstrated an increased fraction of Wnt/TGF-β subclass of tumors and a decreased fraction of the CTNNB1 subclass. In comparison to other etiologies, NASH-related HCC had a considerably higher prevalence of an immunosuppressive cancer field [23]. Moreover, it was also demonstrated that the prognostic liver signature (PLS)-NAFLD predicted incident HCC over up to 15 years of longitudinal observation, while high-risk PLS-NAFLD was associated with IDO1+ dendritic cells and dysfunctional CD8+ T cells in fibrotic portal tracts, with impaired metabolic regulators. PLS-NAFLD was affected by bariatric surgery, lipophilic statins, and the use of IDO1 inhibitors, implicating that it could be utilized in pharmacotherapy and HCC chemoprevention [24].

Interestingly, treatment modalities aiming towards specific genomic alterations form the basis of personalized medicine and constitute the epitome of systemic treatment for many malignancies, but are still not available in HCC. Tools such as liquid biopsy and circulating tumor DNA (ctDNA), even though most studies have not analyzed HCC tissue concomitantly, may be of aid in identifying biomarkers of early diagnosis, response, or resistance to treatment, and their role in HCC represents an ongoing research field [25]. In addition, extracellular vesicles (EVs) or exosomes provide a critical mechanistic way of bidirectional intercellular communication in the TIME of various cancers and it would be very interesting to characterize tumor-derived versus immune-cell-TIME-derived EVs for HCC, in terms of their functionally important genomic, lipidomic, and proteomic cargo [26]. Finally, an integrative analysis of RNA and whole exome sequencing, T-cell receptor (TCR) sequencing, multiplex immunofluorescence, and immunohistochemistry was performed in a cohort of 240 patients with HCC and was validated in other cohorts of 660 patients in total [27]. A 20-gene signature, characterized by high interferon signalling and type I antigen-presenting genes, defined the inflamed class of HCC and was able to capture ~90% of these tumors and was associated with response to immunotherapy. Proteins identified in liquid biopsies recapitulated the inflamed class with an area under the ROC curve (AUC) of 0.91.

## 4. Critical Analysis of Data and Future Perspectives

Taking into account the aforementioned complex molecular omics alongside the heterogeneous cellular landscape of the TIME of HCC, future studies are expected to highlight in a simple way that is practical for clinical use, the hepatic and peripheral blood inflammatory and immunosuppressive tumorigenic function and composition of TANs and TAMs in NASH-related HCC in comparison with other aetiologies and, more importantly, to shed further light on potential prognostic or predictive molecular markers for future immunotherapies targeting TANs and TAMs, acting in synergy with ICIs, in order to overcome resistance and eventually improve the percentage of patients with HCC that respond to treatment. In addition, we underline the significance of predictive biomarkers of response to ICIs in order to (i) enhance the overall survival of patients that are likely to respond to therapy, (ii) reduce the risk of treatment-related adverse effects conveyed through the combination of drugs such as bevacizumab, (iii) maximize efficacious application, and therefore the cost-effectiveness of different treatment modalities, and (iv) characterize the molecular landscape of patients with advanced HCC responding to anti-PD1 therapy and define a novel tool for patient selection in future clinical trials.

All of these data render HCC an oncological diagnosis in which spontaneous immunogenicity is critical for the efficacy of immunotherapy. Although the predictive value of histopathologic assessment is unparalleled, TIME immunogenicity is influenced by density, functional polarization, and distribution of the infiltrate. The diversity of the HCC TIME and the demand for readily applicable biomarkers rather than complex transcriptomics is still challenging, while the ultimate goal of expanding the reach of effective cancer immunotherapy to a wider proportion of patients via clinical stratification of trial participants or targeted testing of novel combinations prognostically modulating adverse traits, such as TANs and TAMs infiltration, is of the utmost importance.
